# Altered gray matter organization in children and adolescents with ADHD: a structural covariance connectome study

**DOI:** 10.1038/tp.2016.219

**Published:** 2016-11-08

**Authors:** K R Griffiths, S M Grieve, M R Kohn, S Clarke, L M Williams, M S Korgaonkar

**Affiliations:** 1 Brain Dynamics Centre, The Westmead Institute for Medical Research, The University of Sydney, Westmead Sydney, NSW, Australia; 2 Sydney Translational Imaging Laboratory, Heart Research Institute, Charles Perkins Centre and Sydney Medical School, University of Sydney, Sydney, NSW, Australia; 3 Adolescent and Young Adult Medicine, Westmead Hospital, Sydney, NSW, Australia; 4 Centre for Research into Adolescents' Health (CRASH), Sydney, NSW, Australia; 5 Psychiatry and Behavioral Sciences, Stanford University, Stanford, CA, USA; 6 Department of Sierra-Pacific MIRECC, VA Palo Alto Health Care System, Palo Alto, CA, USA; 7 Discipline of Psychiatry, Sydney Medical School, Westmead, Sydney, NSW, Australia

## Abstract

Although multiple studies have reported structural deficits in multiple brain regions in attention-deficit hyperactivity disorder (ADHD), we do not yet know if these deficits reflect a more systematic disruption to the anatomical organization of large-scale brain networks. Here we used a graph theoretical approach to quantify anatomical organization in children and adolescents with ADHD. We generated anatomical networks based on covariance of gray matter volumes from 92 regions across the brain in children and adolescents with ADHD (*n*=34) and age- and sex-matched healthy controls (*n*=28). Using graph theory, we computed metrics that characterize both the global organization of anatomical networks (interconnectivity (clustering), integration (path length) and balance of global integration and localized segregation (small-worldness)) and their local nodal measures (participation (degree) and interaction (betweenness) within a network). Relative to Controls, ADHD participants exhibited altered global organization reflected in more clustering or network segregation. Locally, nodal degree and betweenness were increased in the subcortical amygdalae in ADHD, but reduced in cortical nodes in the anterior cingulate, posterior cingulate, mid temporal pole and rolandic operculum. In ADHD, anatomical networks were disrupted and reflected an emphasis on subcortical local connections centered around the amygdala, at the expense of cortical organization. Brains of children and adolescents with ADHD may be anatomically configured to respond impulsively to the automatic significance of stimulus input without having the neural organization to regulate and inhibit these responses. These findings provide a novel addition to our current understanding of the ADHD connectome.

## Introduction

Attention-deficit hyperactivity disorder (ADHD) is the most prevalent developmental disorder, affecting 6–7% of school-aged children and adolescents. It is characterized by developmentally inappropriate symptoms of inattention, impulsivity and hyperactivity, and is often associated with poor academic and social outcomes.^1^ Neuroimaging studies have linked these cognitive control, attention and emotion processing related impairments to functional and structural abnormalities in distributed brain regions including the inferior frontal gyrus, posterior cingulate, caudate nucleus, inferior parietal lobe, amygdala and cerebellum.^2, 3, 4^ The manner in which these regions behave within a network is crucial in understanding these symptoms and has led to the more recent view that ADHD may be best described in terms of aberrance in a number of large-scale functional brain networks such as the default mode network (DMN), ventral attention network (VAN), frontoparietal and limbic networks.^5^ Whether these functional disturbances are also reflected in altered gray matter organization of these large-scale networks still remains to be tested.

Graph theory analysis is a powerful method for quantifying and characterizing brain networks, and has been instrumental in a system level understanding of the brain.^6^ It permits a data-driven exploration of topological organization of large-scale brain networks (that is, connectomes) in a way not previously possible, producing a number of summary metrics that describe the properties of brain networks. For instance, small-world and network efficiency measures describe the balance between information segregation and integration at the global level, while regionally specific properties such as nodal degree demonstrate the contribution of ‘hubs' that facilitate integrative processes.^6^ Previous studies of graph theoretical analysis of whole-brain resting-state functional magnetic resonance imaging (MRI) data have reported that children and adolescents with ADHD exhibit higher local clustering and lower global efficiency compared with Controls. This suggests that children and adolescents with ADHD may have less-optimized topological organization, reflecting a maturational delay in formation of functional networks^7, 8^ (see Cao *et al.*^9^ also for a review including network-specific findings). Cao *et al.*^10^ observed similar findings using white matter probabilistic tractography of diffusion imaging data in ADHD, demonstrating convergence between functional and structural organizational metrics.

To date, however, no study has reported on network measures in participants with ADHD using covariance between regional gray matter volumes. Structurally and functionally connected brain regions tend to show ‘in sync' fluctuations in gray matter volume over time. This phenomenon, known as structural covariance, is theorized to reflect developmental coordination or synchronized maturation between regions of the brain due to mutually trophic influences.^11, 12^ A gray matter network analysis approach using brain volume data derived from T1-weighted images measures this covariance, thus providing a developmental ‘snapshot' of the brain that is complementary to diffusion and functional MRI data.^13^ This is particularly relevant in ADHD, in which cortical maturational delay is one of the leading etiological theories.^14^ This data-driven approach may also shed light on the structural networks and the key hubs mediating specific functional impairments commonly observed in ADHD.

In this study, we use T1-weighted MRI data to parcellate the brain into 92 cortical and subcortical gray matter regions in children and adolescents with and without ADHD. Volumes of these regions were used to map structural covariance networks for evaluation using graph theory. We hypothesized that compared with typically developing Controls, children and adolescents with ADHD would exhibit abnormal network topology. More specifically, we predict higher clustering and lower global integration compared with Controls. Further, we predict that network hubs will differ between these groups predominantly in regions crucial to observed clinical cognitive findings.

## Materials and methods

### Participants

MRI data were drawn from 39 children and adolescents with ADHD scanned at Westmead Hospital (Western Sydney Local Health District) as part of the baseline data collection for the international Study to Predict Optimized Treatment in ADHD (iSPOT-A; protocol described further in Elliott *et al.*^15^). ADHD diagnoses were established by referring clinicians, based on Diagnostic and Statistical Manual of Mental Disorders (4th ed., DSM-IV; American Psychiatric Association, 1994) criteria. Research psychologists confirmed diagnosis and subtype using the parent-rated ADHD Rating Scale IV (ADHD-RS IV^16^), indicated by a score >1 in six or more items in the inattentive and/or hyperactive-impulsive sections of the scale. Twenty individuals with ADHD were determined to have inattentive subtype, while 19 were combined subtype. ADHD participants were aged 8–17 years, fluent in English and stimulant naive (*n*=14) or unmedicated for a minimum of 7 days prior to the time of testing (*n*=25). Referring clinicians and research psychologists used the Mini International Neuropsychiatric Interview for children and adolescents (MINI Kid; Sheehan *et al.*^17^) to exclude any participants with learning disorders, current or past alcohol/drug abuse, or any comorbid axis 1 disorder. Ten participants with ADHD (29%) had comorbid oppositional defiant disorder.

Comparison was made against 30 age- and gender-matched typically developing Controls. Controls were screened for the absence of Axis 1 mental disorders using the MINI-KID and SPHERE-12.^18^ Sample size was based on previous studies investigating whole-brain connectomics using diffusion tensor imaging and resting-state functional MRI in child and adolescent ADHD.^7, 10^ No participants had a history of brain injury, any significant medical condition affecting brain function (for example, epilepsy), or any contraindications for MRI. This study received institutional review board approval from the Human Research Ethics Committee, Western Sydney Local Health District and was conducted according to the principles of the Declaration of Helsinki 2008. All participants (and/or guardian when <16 years) provided written informed consent.

### Image acquisition and preprocessing

MRI data were acquired on a 3.0T GE Sigma HDx scanner (GE Healthcare, Milwaukee, WI, USA) using an 8-channel head coil. Three-dimensional T1-weighted magnetic resonance images were acquired in the sagittal plane using a 3D SPGR sequence (TR=8.3 ms; TE=3.2 ms; flip angle=11° TI=500 ms; NEX=1; ASSET=1.5; frequency direction: S/I). A total of 180 contiguous 1 mm slices were acquired with a 256 × 256 matrix, with an in-plane resolution of 1 mm × 1 mm, resulting in isotropic voxels.

Pre-processing of the T1-weighted images was performed using the VBM8 toolbox (http://dbm.neuro.uni-jena.de), implemented within the SPM8 package (http://www.fil.ion.ucl.ac.uk/spm). First, MRI data sets were visually inspected for artifacts and movement by two independent investigators, and all scans passed an automated quality assurance protocol within VBM8. Specifically, sample homogeneity was visually assessed to identify any outliers who were two or more s.d's of GM volume outside of the sample distribution.

Images were corrected for bias-field inhomogeneity and tissue-classified into gray matter, white matter and cerebrospinal fluid. Study-specific (child/adolescent) tissue probability maps were created using the template-o-matic toolbox,^19^ and implemented during registration to standard space using high-dimensional DARTEL normalization.

Warped tissue-type images were modulated to preserve the volume of a particular tissue using the Jacobian determinants derived from spatial normalization. Seven subjects (5 ADHD, 2 Control) were excluded for motion artifacts that distorted the boundary between segmented gray and white tissue images, leaving a total of 34 ADHD subjects (25 males, 9 females) and 28 typically developing Controls (19 males, 9 females) for analysis. The mean sample homogeneity covariance for the remaining individuals in each group was 0.74, indicating high data quality (that is, minimal artifacts) and no differences in scan quality between groups.

### Defining network nodes for analysis

We referenced the nodes for analysis according to the automated anatomical labeling atlas.^20^ We extracted data for 92 cortical and subcortical regions, defined using the WFU PickAtlas Toolbox.^21^ These regions include the 90 regions defined in previous graph analysis studies of structural correlation networks.^22, 23^ We also included the bilateral cerebellum, as functional imaging studies in ADHD commonly report an altered dysfunction and connectivity associated with this region.^24^ Volumes were extracted from modulated, normalized non-linear GM images (that is, corrected for total brain volume) for each region using MarsBaR.^25^ Supplementary analyses were also conducted using cortical area parcellations from resting-state correlations^26^ to determine that results were not unduly influenced by the number of nodes or parcellation method.

### Construction of structural covariance networks

**Figure 1:**
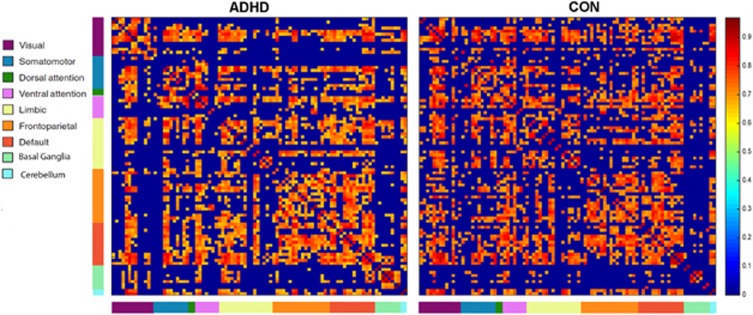
Association matrices for attention-deficit hyperactivity disorder (ADHD) and Control (CON) groups. The right-side color bar indicates the strength of connectivity. These association matrices are thresholded at the minimum network density (36%) in which the networks of both groups are not fragmented and paths exist between each node and every other node. Correlations below this threshold are set to zero. Brain regions are organized relative to intrinsic functional connectivity networks,.^27^ Each region is presented as a left/right pair in the association matrix.

Gray matter volumes of all 92 anatomical regions for each individual were used to construct structural covariance networks, in preparation for graph theory analysis. To compute a structural covariance network for each group, a 92 × 92 association matrix, R, was generated. To enhance clarity, regions were organized relative to intrinsic functional connectivity networks^27^ (Figure 1). These networks were visual, somatomotor, dorsal attention, ventral attention, limbic, frontoparietal, default mode, as well as the basal ganglia and cerebellum (see Supplementary Table 1, Supplementary Material for classifications). We note that the network analyses described below were completely data driven across the whole brain, with no *a priori* bias of these network definitions in the analysis. Each entry, *r_ij_*, was defined as the Pearson correlation coefficient between gray matter volume measures of regions *i* and *j*, across participants.^22, 28^ A binary, undirected adjacency matrix was derived from each association matrix, whereby each coefficient was considered 1 if it was greater than a specific threshold and zero otherwise. The diagonal elements of the association matrix represent self-connections and were therefore excluded from analysis. Due to the methodological challenges in analyzing and comparing weighted networks,^29^ a graph was constructed with 92 nodes, with a network degree of E equal to number of edges (links) and a network density (cost) representing the fraction of present connections to all possible connections.

### Graph theoretical analyses

Graph theoretical analyses were performed on the interregional covariance matrices using the Brain Connectivity Toolbox (http://www.brain-connectivity-toolbox.net/) and the Graph Analysis Toolbox.^22^ BrainNet viewer^30^ was used for visualization of regional analyses.

#### Global network analyses

To allow comparison of global network properties between groups and avoid biases associated with using a single threshold, the association matrices were thresholded at a range of network densities, in 0.02 steps (*D*_min_: 0.02:*D*_max_). The minimum density was that at which the networks of both groups were not fragmented and paths exist between each node and every other node. The maximum density chosen was 0.50, as after this threshold the graphs become increasingly random.^22^

At each of these thresholds, we calculated the following global network measures: (1) the characteristic path length (the mean number of connections on the shortest path between any two regions in the network and is a measure of network integration); (2) the clustering coefficient (quantification of the probability that two nodes connected to an index node are also connected to each other and is a representation of network segregration); and (3) small-worldness (the balance between local segregation and global integration).

To evaluate these topological measures, they were benchmarked against corresponding mean values of a null random graph. We generated 20 null networks from covariance matrices that were matched to the distributional properties of the observed covariance matrix using the Hirschberger–Qi–Steuer algorithm.^16^ ADHD and Control groups were also compared on non-normalized global measures.

#### Regional network analyses

Local nodal characteristics of individual network regions were also examined using the following measures: (1) nodal degree, which is the number of connections that a node has with the rest of the network, and (2) betweenness centrality, which is a measure of the number of shortest paths that traverse a given node and is used to detect nodes that are highly central for important anatomical or functional connections.

Hubs are nodes that are critical for efficient communication in a network, and have a key role in the regulation of information flow.^6^ A node was considered to be a hub if its degree was at least one s.d. higher than the mean network degree.^23^ Nodes were normalized by the mean network betweenness and degree of each group before between-group comparisons.^22, 23^

### Comparing network measures between groups

A nonparametric permutation test with 1000 repetitions was conducted to test the statistical significance of ADHD-associated differences in global and regional network topologies. In every permutation, each participant was randomly reassigned to the ADHD or Control group such that each group maintained their original number of subjects. We subsequently obtained an association matrix for each randomized group, thresholded at a range of network densities, which led to a binary adjacency matrix at each threshold. Network measures were calculated for all binary adjacency matrixes at each density. Differences in network measures between randomized groups were then calculated, resulting in a permutation distribution of difference under the null hypothesis. Differences in ADHD and Control network measures were placed in the corresponding permutation distribution and two-tailed *P*-values were calculated based on their position.^31^ This nonparametric permutation approach inherently accounts for multiple comparisons across densities.^32^ False discovery rate-corrected *P*-values were reported for all between-group differences to account for multiple comparisons (three global measures).

In addition to comparing global network measures at every density, functional density analyses were performed to make the between-group comparison less sensitive to the thresholding process. As network metrics were calculated across each of the specified densities (*D*_min_:0.02:0.50), they were represented by a curve depicting change in network metric as a function of network density. Permutation tests were subsequently applied to the functional density analyses output to determine whether there were significant group differences. As comparison at every network density would result in a large number of comparisons (number of densities × number of regions of interest), the group comparison of regional measures was based on the functional density analyses result.^22^ False discovery rate-corrected *P*-values were reported for regional differences to account for multiple comparisons across the multiple nodes of interest.

## Results

ADHD and Controls did not differ in age (ADHD, *m*=13.28±2.7; Controls, *m*=13.33±2.7), *t*(60)=−0.07, *P*=0.95, or gender (ADHD, 25 males (64.1%); Controls, 19 males (67.9%)), *χ*^2^(1,*N*=62) =0.24, *P*=0.78. Using the ADHD-RS, mean total symptom severity was 34.4±7.4.

### Global network analyses

Within each group, the minimum density in which all nodes became connected in the network was 0.36. Greater than 93% of all regional covariances were significant at *P*<0.05 in the matrices of both groups.

**Figure 2:**
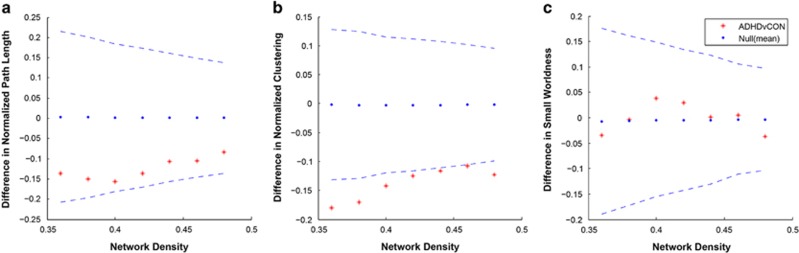
Differences between attention-deficit hyperactivity disorder (ADHD) and Control (CON) groups in global network measures across a range of network densities. (**a**) Normalized path length, (**b**) normalized clustering coefficient, (**c**) normalized small-worldness index. Asterisks represent the difference between ADHD and Control groups, with dashed lines indicating 95% confidence intervals (CIs). Asterisks outside of the CI indicate densities in which the difference is significant at *P*<0.05. Positive values show ADHD<CON and negative values show ADHD>CON. ADHD exhibited a significant increase in normalized clustering coefficient relative to the Control group at all network densities.

The ADHD group exhibited an increased clustering coefficient relative to the Control group (Figure 2), a finding that occurred across all measured network density thresholds (minimum density, corrected *P*=0.017). The functional density analysis confirmed that this result was not driven by differences in correlation strengths in regional gray matter volumes that would make the analysis less sensitive to thresholding, corrected *P*=0.017. There were no significant differences at any network density thresholds between ADHD and Control groups for characteristic path length or small-worldness. Small-worldness was >1 across all network densities in both groups (Figure 1, Supplementary Materials). Comparison of non-normalized network measures also indicated increased clustering in ADHD relative to Controls (corrected *P*=0.028), and no difference in path length or small-worldness.

### Regional network analyses

**Figure 3:**
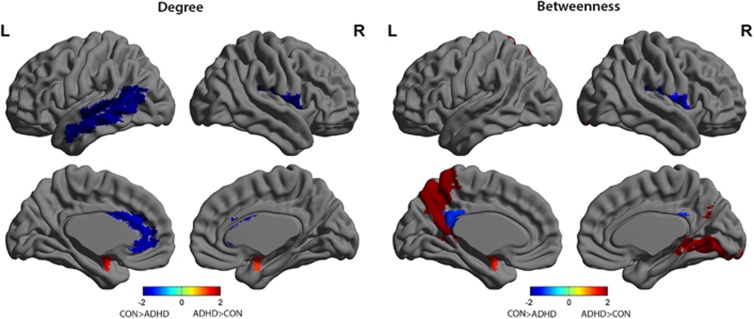
Differences between attention-deficit hyperactivity disorder (ADHD) and Control (CON) groups in regional degree and betweenness. Regions with significant group differences in regional degree (left) and betweenness (right) for networks thresholded at minimum density of full connectivity, overlaid on ICBM152 surface template. The color bar represents log(1/*P*-value). Hot colors indicate regions with higher degree or betweenness in ADHD compared with Controls, while cold colors indicate regions with higher degree or betweenness in Controls compared with ADHD. ADHD had greater nodal degree in the bilateral amygdalae, and reduced degree in left (L) anterior cingulate, L mid temporal pole and right (R) rolandic operculum. Nodal betweenness was increased in ADHD compared with Controls in L amygdala and precuneus, and R lingual gyrus, but reduced in L posterior cingulum and R rolandic operculum.

Group comparison of regional network measures highlighted significantly greater nodal degree for the bilateral amygdalae for the ADHD group relative to Controls (left, corrected *P*=0.027; right, corrected *P*=0.048). The ADHD group had significantly reduced nodal degree compared with Controls in the left anterior cingulate (corrected *P*=0.016; DMN), left mid temporal pole (corrected *P*=0.008; DMN) and right rolandic operculum (corrected *P*=0.011; VAN). Nodal betweenness was increased in ADHD compared with Controls in the left amygdala (corrected *P*=0.04) and precuneus (corrected *P*=0.01; DMN), and right lingual gyrus (corrected *P*=0.013; visual network), but reduced in the left posterior cingulum (*P*=0.047; DMN) and right rolandic operculum (corrected *P*=0.025; VAN; Figure 3)

**Figure 4:**
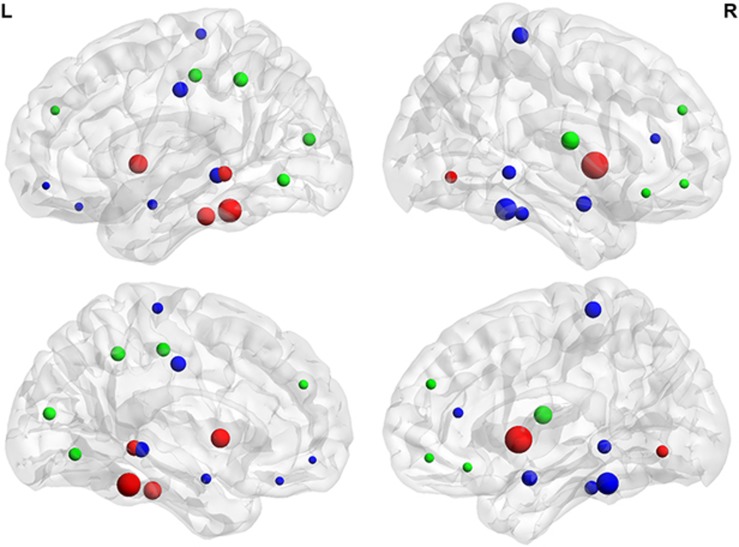
Summary of network hubs. Red nodes denote network hubs present in both attention-deficit hyperactivity disorder (ADHD) and Control (CON) groups. Blue nodes are unique to the ADHD group while green nodes are unique to CON group. The size of the sphere represents the degree of the corresponding brain region. Network hubs that were present in both ADHD and CON were L orbital inferior frontal gyrus, L fusiform gyrus, bilateral insula, R lingual gyrus and L inferior temporal gyrus. Hubs unique to ADHD were bilateral amygdalae, R anterior cingulate, L mid cingulate, L medial orbito frontal cortex, R fusiform, bilateral paracentral lobules, L rectus, R inferior temporal gyrus and bilateral mid temporal gyri. Hubs unique to CON were R orbital inferior frontal gyrus, R orbital medial frontal gyrus, bilateral superior medial frontal gyrus, L lingual gyrus, L mid occipital gyrus, L inferior parietal lobule, L postcentral gyrus and R rolandic operculum.

The hub analysis revealed 15 network hubs in the Control group and 18 in the ADHD group (Figure 4). Hubs present in the ADHD group that were not present in the Controls included the bilateral amygdala, paracentral gyrus, right cingulate cortex and bilateral temporal cortex. Hubs that were unique to the Control group were the right orbital inferior frontal gyrus, right medial orbital frontal gyrus, bilateral medial superior frontal gyrus, left lingual gyrus, left mid occipital gyrus, left inferior parietal gyrus, left postcentral gyrus and right rolandic operculum. Hubs that were present in both Control and ADHD networks were the bilateral insula, left inferior orbital cortex, fusiform gyrus and left inferior temporal lobe.

When using a different parcellation method, there was a replication of the finding that the amygdalae were hubs in ADHD but not Controls (see Supplementary Materials for full results using alternate parcellation method).

## Discussion

To the best of our knowledge, this is the first study to investigate gray matter volume covariance networks of children and adolescents with ADHD. As hypothesized, there was evidence of altered large-scale brain network organization in ADHD relative to age- and gender-matched typically developing Controls. Specifically, ADHD participants exhibited greater segregation in global network organization, indexed by significantly increased clustering. At a regional level, the ADHD group had significantly greater bilateral amygdalae participation, as well as reduced hub strength within regions of the DMN and VAN networks. These differences in brain network hubs align with the behavioral disturbances commonly observed in ADHD such as emotional dysregulation and poor executive control.^33, 34, 35^

Although, functional MRI or diffusion tensor imaging measures have been widely used to evaluate brain connectivity, covariance of morphological metrics derived from anatomical MRI scans have been shown to extract information about brain connectivity.^11, 12^ At least 40% of anatomical covariance across the cerebral cortex has also been shown to converge with white matter connections.^36^ As with functional connectivity, anatomical correlation does not depend on the existence of a direct fiber connection between regions and could arise from indirect connections that are mediated by another source. In that respect, network connectivity based on anatomical covariance measures could have greater concordance with functional connectivity. Covariance matrices derived from anatomical measures is amenable to network analysis in a manner analogous to functional network analysis with graph properties of networks derived from anatomical covariance measures found to change with normal development as well as with disease.^13, 23^

### Increased clustering in network topology

Convergent evidence from graph theory measures in other imaging modalities supports our finding of increased global clustering or network segregation in children and adolescents with ADHD. We did not however observe any significant differences for measures of integration and small-worldness between the groups. Previous studies using resting-state fMRI^7^ and white matter probabilistic tractography^10^ have found increased local segregation, combined with reduced distribution or integration of processing in ADHD participants relative to Controls. Using delta frequency electroencephalogram, Ahmadlou *et al.*^8^ also found higher clustering within the left hemisphere. Diversity of imaging modalities, methodology and sample characteristics may have contributed to any discrepancies in these quantitative global network measures; however, all studies show an overall phenomenon of less-optimized networks in child and adolescent ADHD.

Healthy neurodevelopment is thought to follow a local to distributed organizational principle.^37, 38^ Late childhood and early adolescence are particularly important times due to synaptic pruning of gray matter occurring in conjunction with increased myelination of white matter tracts.^39^ This improves neural efficiency and allows for rapid information transfer between distant brain regions, which is important for the integrative nature of many higher-order cognitive processes that develop during this critical period.^38^ As such, greater localized segregation in ADHD relative to age-matched Controls is supportive of the notion of maturational delay in whole-brain network organization. Although longitudinal studies have been instrumental in mapping regions of maturational lag in cortical thickness and surface area in children and adolescents with ADHD,^14^ our data support a shift in focus from variance within localized gray matter morphometry to more large-scale alterations happening within distributed networks and their organization. Future longitudinal studies will be required however to determine whether this network topology is merely delayed or whether it represents aberrance in the neurodevelopmental trajectory.

### Differences in regional network measures in ADHD

Nodes with high degree and betweenness typically suggest highly interactive regions that likely participate in more functional interactions.^6^ These nodes may also be considered ‘hubs' if they have greater degree relative to the average of the network. Our hub analysis revealed a slightly greater number of hubs in the ADHD cohort relative to Controls, with hubs distributed across the limbic, DMN, VAN, somatomotor and frontoparietal networks.

A direct group comparison revealed that the amygdala had greater network influence in the ADHD cohort relative to Controls as reflected by greater nodal degree bilaterally and increased betweeness in the left amygdala. This is also reflected as the amygdalae were hubs regions for the ADHD groups but not for the Control group. This increased nodal degree might be a basis for amygdala-related limbic functional disruptions observed in ADHD.

Children and adolescents with ADHD often exhibit symptoms of increased emotional sensitivity,^40, 41^ higher rates of depression and anxiety^42^ and deficits in identifying threat-related emotional expressions accompanied by an inability to differentiate emotions at a neural level.^35^ Amygdala hyperactivation and abnormalities within bottom-up processing networks have been reported during emotion and reward processing in ADHD (see Shaw *et al.*^41^ for a review), as well as abnormalities in amygdalar dopamine receptor density.^43^ Increased amygdala activation and amygdala-lateral prefrontal cortex connectivity during viewing of fearful faces have been posited to underlie increased emotional sensitivity in adolescents with ADHD.^44^ Emotion dysregulation-related associations with resting functional connectivity between the amygdala and the rostral anterior cingulate have also been reported in children with ADHD.^45^ Our results provide new evidence that there may be an anatomical network disorganization surrounding the amygdala in ADHD.

In addition to emotion dysregulation, hallmark symptoms of ADHD are impaired attention and response inhibition. We found significantly reduced nodal degree in ADHD relative to Controls in the anterior cingulate cortex. A number of studies have found dysfunction of the anterior cingulate cortex in ADHD,^46^ which has been identified as part of the so-called ‘rich club'—a group of high-degree nodes that tend to be densely interconnected and have a key role in global information integration.^47^ This supports Ray *et al.*,^48^ who recently reported that individuals with ADHD exhibit reduced connectivity within rich-club networks. It is an important node within multiple networks, including the DMN, frontoparietal, salience and limbic networks. The frontoparietal network has an essential role in top-down regulation and enabling goal-directed processes, which is particularly relevant to the motivational and cognitive theories of ADHD.^49^ The cognitive division of the anterior cingulate specifically has a role in attentional processing by modulating stimulus selection and mediating response selection.^50^ The DMN on the other hand is thought to reflect task-independent introspection or ‘mind wandering' and has an antagonistic relationship with networks engaged during conditions demanding attention.^51^ As such, default mode interference may also contribute to attentional dysregulation.^52^

A number of recent studies have found dysregulation within the DMN and its connectivity with task-positive networks in ADHD.^53, 54, 55^ In the current study, the ADHD network exhibited decreased degree and/or betweenness compared with Controls for three nodes within the DMN: the left anterior and posterior cingulate and mid temporal pole. In contrast, the ADHD group also exhibited increased betweenness for the left precuneus relative to Controls. Increased betweenness suggests that this prominent DMN node participates with disparate parts of the whole-brain network (more so than in Controls). Given the antagonistic relationship between the DMN and task-positive networks, this may support the view that default mode interference has a role in attentional impairment.

Reduced nodal degree and betweenness was observed in the right rolandic operculum for the ADHD group. This region is considered to be an important language hub^56^ and is also part of the VAN, which monitors for salient stimuli and reorients attention when appropriate.^57^ Reduced interactions of this region with other parts of the brain may contribute to the increased prevalence of language dysfunction in ADHD children compared with typically developing Controls.^58^ This region is not typically associated with ADHD pathology. In addition, we did not find the typically reported abnormalities within fronto-striatal circuits.^2^ This, however, may be a function of the structural covariance methodology and shines light on difference aspects of ADHD pathology.

Overall the findings suggest that ADHD is characterized by a disruption to the normal anatomical organization of neural circuits at the whole-brain level. This disruption involves a greater reliance on subcortical connections specifically with the amygdala, a region at the core of appraising emotional significance, at the expense of cortical hubs important to the dynamical regulation of multiple neural circuits, such as the anterior and posterior cingulate.^46^ A shift toward more subcortical but fewer cortical connections may imply that the brains of children and adolescents with ADHD are likely to be anatomically configured to respond impulsively to the automatic significance of stimulus input without having the neural organization to regulate and inhibit these responses. Although not tested directly in this study, this fits with behavioral symptoms observed in previous studies.^41^ Analysis of the organization and functioning of entire networks contributes to a more complete picture of neuropathology and has tremendous potential for providing new insights that would have previously been missed using a regionally focused framework. Enhanced knowledge of the mechanisms underlying ADHD through new methodologies provides greater diagnostic precision, which has potential for informing animal models of ADHD and identifying new treatment targets.

### Limitations

This study has several limitations. First, our findings are based on cross-sectional observations, which limit how we can interpret maturational lag in network topology. Future longitudinal studies will be required to determine whether network topology is merely delayed or whether it represents aberrance in the neurodevelopmental trajectory. Second, we parcellated brains using the automated anatomical labeling atlas, which divides the cortex and subcortical structures into regions based on anatomical landmarks. This may be relatively arbitrary with respect to functional areas and nuclei. Nonetheless, our supplementary analyses using 347 nodes derived from functional connectivity reproduced the key regional finding of hubs distributed predominantly within ventral emotion and reward centers (Supplementary Figure 2). Although organizational principles of structural brain networks seem to be largely independent of parcellation approach, it may significantly modulate quantitative measures of these principles,^59^ which we saw in our inability to replicate the finding of increased clustering with the different parcellation method. We also found an opposing effect in our group comparison of nodal degree in the left anterior cingulate when using a different parcellation approach. This highlights the importance of parcellation approaches when interpreting data and comparing across studies. Third, our structural covariance approach is confined to a population-based definition of connectivity, which restricts direct correlation of network measures with individualized clinical and behavioral metrics. Recent findings show that rich-club connectivity and higher global network integration are associated with higher intellectual abilities in typically developing children.^60^ We did not have a reliable measure of IQ in this sample, therefore we cannot determine whether our results were mediated by group differences in IQ. Finally, our sample size is modest considering that ADHD is quite a heterogeneous disorder. Long-term effects of previous medication use and oppositional defiant disorder comorbidity are additional factors that may have increased biological heterogeneity within our sample and potentially impacted structural covariance.^61, 62^ It will be important to replicate these findings in independent cohorts with larger sample size and more homogenous, well-characterized populations.

Despite these limitations, our findings advance our understanding of the ADHD connectome. The findings demonstrate that ADHD is characterized by disruptions to both the global and local organization of brain morphometry. Structural covariance metrics offer promise for helping to refine the mechanistic understanding of ADHD and for potential treatment targets.
