# Supplementary figures and images for: Altered gray matter organization in children and adolescents with ADHD: a structural covariance connectome study
Source: Transl Psychiatry. 2016 Nov 8;6(11):e947–. doi: 10.1038/tp.2016.219 (PMC5314130; doi:10.1038/tp.2016.219)

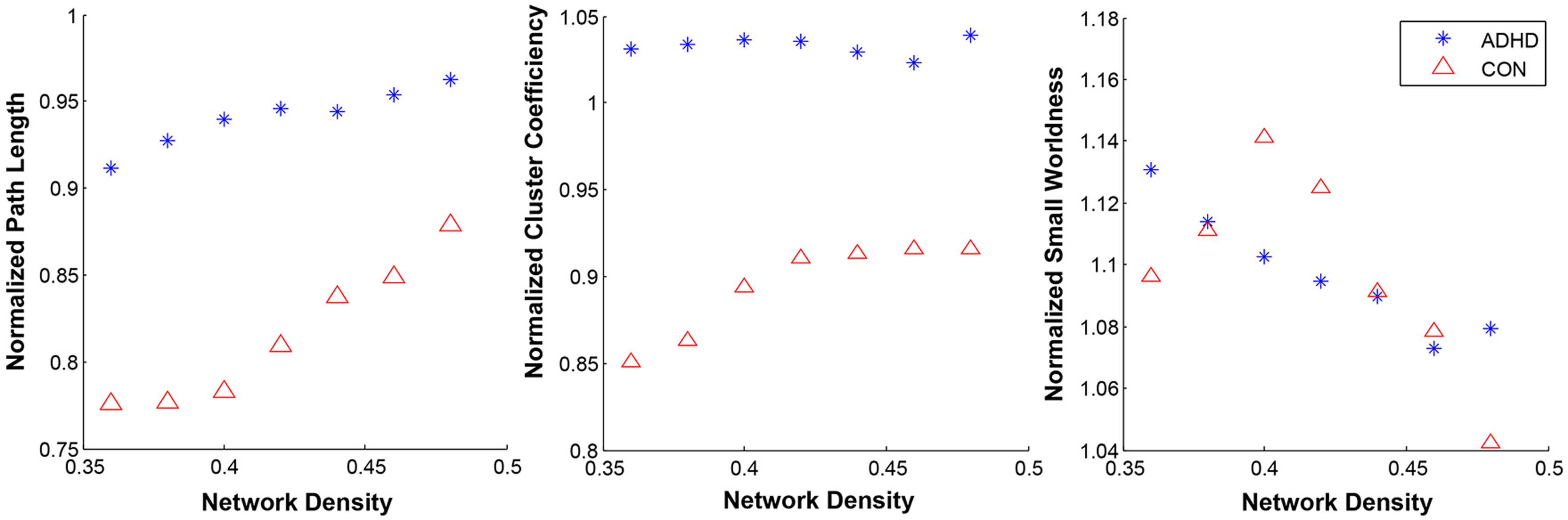

Supplement: Supplementary Figure 1 [file tp2016219x1.tif]

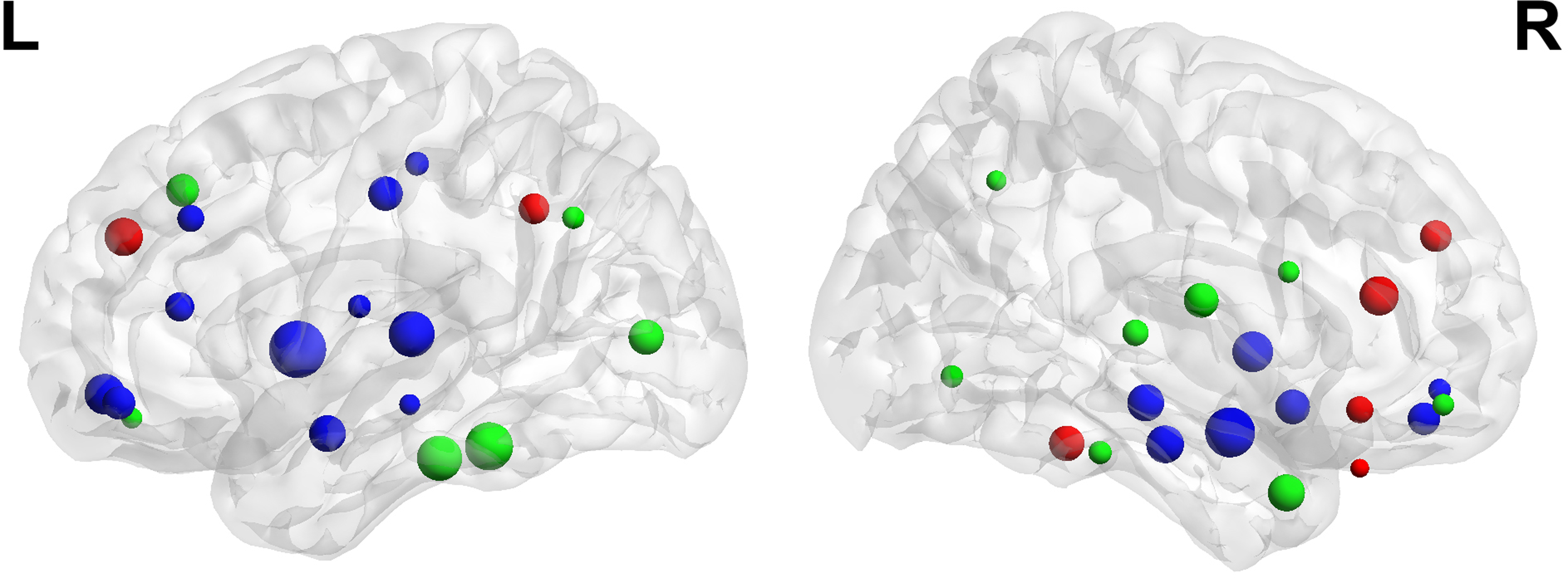

Supplement: Supplementary Figure 2 [file tp2016219x2.tif]
