# Supplementary material for: Altered gray matter organization in children and adolescents with ADHD: a structural covariance connectome study
Source: Transl Psychiatry. 2016 Nov 8;6(11):e947–. doi: 10.1038/tp.2016.219 (PMC5314130; doi:10.1038/tp.2016.219)
Supplement: Supplementary Table 1 [file tp2016219x3.docx]

Table 1. AAL regions overlayed with approximate functional networks

| **Network** | **AAL Region** |
| --- | --- |
| VISUAL | Calcarine |
|  | Cuneus |
|  | Fusiform |
|  | Lingual |
|  | Occipital Inferior |
|  | Occipital Mid |
|  | Occipital Superior |
| SOMATOMOTOR | Heschl |
|  | Paracentral Lobule |
|  | Postcentral |
|  | Precentral |
|  | Temporal Superior |
| DORSAL ATTENTION | Parietal Superior |
| VENTRAL ATTENTION/ | Insula |
| SALIENCE | Rolandic Operculum |
|  | Supplementary Motor Area |
|  | Supra Marginal |
| LIMBIC | Amygdala |
|  | Frontal Medial Orbital |
|  | Hippocampus |
|  | Olfactory |
|  | Para Hippocampal |
|  | Rectus |
|  | Temporal Pole Mid |
|  | Temporal Pole Superior |
| FRONTOPARIETAL | Angular |
|  | Frontal Mid |
|  | Frontal Mid Orbital |
|  | Cingulum Mid |
|  | Frontal Inferior Operculum |
|  | Frontal Inferior Trigeminal |
|  | Frontal Superior |
|  | Frontal Superior Orbital |
|  | Parietal Inferior |
| DEFAULT MODE | Cingulum Anterior |
|  | Cingulum Posterior |
|  | Frontal Inferior Orbital |
|  | Frontal Superior Medial |
|  | Precuneus |
|  | Temporal Inferior |
|  | Temporal Mid |
| BASAL GANGLIA | Caudate |
|  | Pallidum |
|  | Putamen |
|  | Thalamus |
| CEREBELLUM | Cerebellum |

*NB. As these are different parcellation methods, they do not overlay perfectly. Some AAL regions fall within multiple functional networks. This table is an approximation for aiding general interpretation.
