# Supplementary material for: Altered gray matter organization in children and adolescents with ADHD: a structural covariance connectome study
Source: Transl Psychiatry. 2016 Nov 8;6(11):e947–. doi: 10.1038/tp.2016.219 (PMC5314130; doi:10.1038/tp.2016.219)
Supplement: Supplementary Information [file tp2016219x4.docx]

**Supplementary analyses**

Supplementary analyses were also conducted using cortical area parcellations from resting-state correlations (Gordon *et al*, 2016) to determine that results were not unduly influenced by the number of nodes or parcellation method. These 333 regions were combined with 14 regions from the Harvard-Oxford sub-cortical atlas (Desikan *et al*, 2006) to provide full brain coverage with 347 regions. Regional results are reported with their corresponding aal labels for comparison with primary analyses. Due to the large number of regions, hubs were defined as those with the top 10% nodal degree for each group.

*Global Network Analyses*

Within each group, the minimum density in which all nodes became connected in the network was 0.32. There were no group differences in clustering coefficient, characteristic path length or small worldness.

*Regional Network Analyses*

Group comparison of regional network measures revealed significantly greater nodal degree for the ADHD group relative to controls in the left mid temporal lobe, anterior and mid cingulum, insula, mid frontal gyrus, frontal medial orbital, and right supplementary motor area, insula and nucleus acumbens. ADHD nodal degree was reduced in the left superior occipital lobe, mid frontal, frontal superior orbital, and inferior occipital lobe and right supplementary motor area, precuneus, frontal inferior trigeminal, mid temporal pole, superior frontal gyrus.

Hubs present in the ADHD group that were not present in the controls included the bilateral amygdala, left anterior and mid cingulum, postcentral gyrus, and rolandic operculum, and right insula, olfactory, parahippocampus and hippocampus. Hubs that were unique to the control group were the left angular gyrus, calcarine, inferior temporal lobe, frontal superior orbital, fusiform and superior frontal gyrus, and right precuneus, lingual, frontal mid orbital, temporal inferior operculum, frontal inferior operculum, mid temporal pole and superior temporal lobe. Hubs that were present in both control and ADHD networks were the left precuneus, superior temporal, insula, and hippocampus, and right anterior cingulate, right rolandic operculum, frontal superior orbital, and fusiform gyrus. Hubs are presented in supplementary figure 2.

**Supplementary Figure Legends**

**Figure 1**. **Changes in global network measures as a function of network density.** Normalized path length, normalized cluster coefficiency and normalized small worldness of ADHD and control (CON) groups.

**Figure 2. Summary of network hubs using a functionally-derived parcellation method.** Red nodes denote network hubs present in both attention deficit hyperactivity disorder (ADHD) and Control (CON) groups. Blue nodes are unique to the ADHD group while green nodes are unique to CON group. The size of the sphere represents the degree of the corresponding brain region.
